# Ovi Gallinæ Pellicula

**Published:** 1893-06

**Authors:** Samuel Swan

**Affiliations:** New York


### OVI GALLINÆ PELLICULA, OVI MEMBRANI—VESICULA PURKINGE.

SAMUEL SWAN, M. D., NEW YORK.

The above preparations of the egg membranes were partially proved in 1883, and have been frequently used by the writer and others and with great success.

*Sensorium.*—Dizziness and fear of falling, and catching her breath when descending stairs. Walking on anything narrow that is raised above the level of the ground causes the same feeling.

*Mental Symptoms.*—Exceedingly depressed and hopeless.

Hopeless, tearful, inert, mental condition.

Fretful and discontented, because menstrual flow was not more profuse.

Melancholy and down-hearted.

Profound states of melancholy, without any known cause, which pass away quickly like the cloud after a shower, and everything appears bright and cheerful.

*Head.*—Occipital headache.

Aching pain from back of left ear, extending downward over the shoulder half-way from neck; in the afternoon of second day. Similar pain next day from right, extending to the arms.

Fullness of the head with great pressure outward in mastoid region, with headache in occiput, cured.

*Eyes.*—Sharp, darting pains in left eye through to occiput; very violent, compelling her to press the eye to prevent its leaving the socket, which it seemed as if it would do. (Ballard.)

Sensation of a shade over the eyes.

Sunken eyes, haggard look of face.

*Nose.*—Sensation of severe cold, sneezing catarrh, cracked lips (proving).

Severe cold came on with an unusual flow of mucus, which filled three or four handkerchiefs in a day. It caused a tightness of the chest, a great oppression and violent cough. It passed off suddenly.

Catarrh, with hard clinkers, hawked backward, and which invariably shoot down the throat; at ten A. M., three P. M., and ten P. M.

*Face.*—After a dose of CM (which relieved), for a distress in epigastrium, there followed an eruption of small pustules on forehead and chin.

Slight soreness and stiffness of the jaw, which passed off in a few hours; third day.

Looks haggard; sunken eyes; skin discolored—dark.

Sallow complexion—cured.

*Mouth.*—For several days preceding menstruation an exceedingly offensive breath, which when inhaled by a friend seemed to pass down through her, direct to the womb. This continued till menses ceased.

Exceedingly acid saliva (after CM).

*Throat.*—Sore throat every night on left side, as if it had been scalded.

Miss P. took Ovi-gall-p.$^{cm}$. In two days had sore throat, with sensation of a lump on left side low down causing cough; the throat looked inflamed; tonsils swollen, but no indications of ulceration or patches.

Sensation of dryness causing cough. Tonsils remained inflamed on one side. Kalmia relieved.

*Stomach.*—Nervous dyspepsia; low spirits; very weak; sleeplessness, occurring every week.

Sensation of a hard ball or lump in the epigastric region, extending from the lower end of the sternum through to the back, as if she had swallowed a whole potato, and it had lodged there—and with the above was severe pain in the sacral region. Relieved with CM.

Burning in pit of stomach with heaviness and weight; cured.

A very sharp pain passed from epigastric region in a direct line toward the left ovarian region. This was repeated three or four times during the day—each time it was only momentary.

Vomited three gall stones.

*Abdomen.*—Pain in the right hypochondrium, passing into the stomach, with a tendency to diarrhœa; very weak and great dread of motion.

Pain in the liver under the short ribs, exceedingly severe. Gave Ovi-gall-p.$^{emm}$ and the pain immediately passed down to the right knee, then soon passed off; left a weakness after extra exercise.

Severe pain in the left groin, extending to her left foot, caused by over-exertion. In an hour after taking a dose, pain all left and did not return, although she has since taken unusual exercise.

For the past twelve years, at menstrual period the abdomen has been greatly distended. Since taking Ovi-gall-p. there has been no distention at that period.

After menstruation has ceased, cannot bear any pressure on her waist, has to take off her corsets and suspend her clothes from the shoulders.

The night before the last menstruation, she had distention of the abdomen for about two hours, as if it would burst.

Had a pain in left lower abdomen and with it a sensation as if something within turned or rolled over.

(Query, was it natural version of the womb?)

Left side of abdomen feels very sore and occasionally sharp pains dart through it; also a bearing down as if a ten-pound weight were hanging to the womb.

Two days after menstruation complained of sore, aching pains in abdomen. Said the suffering was so great that at times she could hardly stand. Soreness was not increased by external pressure. This continued for one day and then ceased. There was no return of the distention or pains in left side, groin, or limbs.

Kate M. took a dose of CM at night; had pain in lower abdomen, worse on the side she was lying on, and in the sacrum when lying on the back.

A very sharp pain passed from epigastric region in a direct line toward left ovarian region. The pain was momentary and was repeated at intervals three or four times during the day.

Tired feeling in both groins, especially in the left, caused by walking.

Aching, deep inside (three inches) over the left hip bone. This pain disappeared during menstruation and reappeared when menstruation ceased.

*Anus and Rectum.*—Burning in anus after hemorrhage from rectum.

### Stools.

During twelve years has been constipated, going a week or more or having partial movements every four or five days. Since taking Ovi-gall-p. her bowels have moved regularly, except one or two days.

While riding in the cars sudden hemorrhage from the rectum —bright blood. A severe pain followed the discharge across the sacrum. Menses came on next morning.

The day the menses came on had another discharge of blood from the bowels, followed by burning at the anus.

During menstruation sensation as of prolapse of the rectum, which, however, was not the case.

Twenty-four hours after menses commenced had hemorrhage of the bowels as before, but unaccompanied by pain.

Stool mixed with a sand-like substance that fell heavily to the bottom of the vessel, and was voided with great pain.

Diarrhœa, with cutting, colic pains—was icy cold—in a collapsed state. Proving.

Increased peristaltic action of the bowels removing the obstructions that had lodged in the convolution of the intestines *after CM.* Nineteenth day found her clothes wet, and on examination found she had hemorrhage from the rectum; felt miserable all over. At three P. M. next day menses came on, nine days too soon.

*Disorder of Bowels.*—Diarrhœa sometimes mixed with long strips like corrugated skin, most often accompanied with cutting, colic pains. Often three days without a movement.

*Chronic Constipation.*—Stool irregular from five to eight days.

*Diarrhœa* at night, with griping pain in epigastric region, and particularly in pit of stomach, and griping, crampy pains in the bowels. Characteristic, griping cramps, and diarrhœa coming suddenly.

(Note).—This came on four days after taking the drug, and continued four days and then ceased.

*Urine.*—During the proving urine natural, density 20.

Every time she sneezes or coughs the urine spurts out since menstruation ceased.

Urine scalds the vulva, especially on the right side, which itches intensely.

Urine dark yellow-red (after CM.).

*Female Sexual Organs* (external).—Increased pruritus, attended by more than usual swelling of the parts, and with burning of a spasmodic character.

*Vagina.*—Watery, offensive leucorrhœa. Scalding leucorrhœa before menses. Fullness in vagina, hindering walking (perhaps prolapsus).

Since menses ceased has had fluor albus, white, cream-like in consistence, preceded by sharp pains in the womb as during menses. It occurs every morning, whereas the last menses were every other day; has never had anything like it before, except slightly after great fatigue.

Albuminous leucorrhœa in a blonde, æt. twenty-two. Has had it since a child; menses regular, colorless, painless. Gave one dose Ovi-gall-p.$^{cmm}$. Next morning had healthy pains and a flow of bright red blood.—Dr. Boardman.

UTERUS.—Menstrual flow which had ceased returned after one powder CMM. Bearing-down pains in uterine region, with measle-like eruption, and great debility.

Hurries up retarded menses. Given for retarded menses, it produced an abortion in twelve hours, a three-months' fœtus, not previously suspected. At full moon, being nineteen days after taking the drug (30th) and nine days before menstrual period, after three P. M. went to stool and had a natural passage; two hours after felt very wet and about ten P. M., being the first opportunity for examination, found there had been a profuse hemorrhage from the rectum. During the evening had a severe pain across the lower part of back. Next morning at three A. M. menses appeared and during the morning flowed profusely, with pain in left side. Three days after the regular menses had ceased was suddenly seized with strange feelings, a whirling sensation in the head, a sense of depression, weak knees, a giving-away feeling most in the lower limbs, particularly the knees. Immediately there was a rush of blood from the womb, which flowed freely about an hour, then ceased; as the flow appeared the other symptoms passed away, and there was no fullness of the abdomen, neither was there any pain, only a downward pressing.

*Dragging* down as if prolapse of uterus would occur.

Stitches in left ovary day and night, running down into vagina; menses scanty.

Before menses, fluor albus, scalding.

Before menses, was awakened at one A. M. by a pushing-down sensation in lower abdomen as if the blood would instantly rush out in torrents, with enormous distention of abdomen, but no flow came till the following night at one A. M.—natural color, scanty, and in twenty-four hours had ceased altogether. Menstrual flow every other day, preceded the day before by a pushing pressure downward.

*Menses* on time, bright but very scanty; continued twenty-four hours. In two days after menses had ceased she had soreness and aching pains in the abdomen, very severe, not aggravated by pressure; continued one day.

Mrs. P. Menses on time; first day labor pains—a pushing downward in the womb. Passed large masses of clots; never had anything like it before; after Ovi-gall-p.$^{cmm}$.

Albuminous leucorrhœa in place of menses. Cured with

Ovi-gall-p.$^{cmm}$. Next menstruation bright red and more abundant flow; continued five days.

Menses preceded by headache, alternating with pain in left ovarian region; flow free but attended with almost unendurable pains; continued all day (proving).

*Severe* prickling pains in neck of womb, apparently in os; at the same time a pressure downward, attended with intense aching, and a desire to keep the thighs wide apart.

Menses very scanty, natural color. The last part of the flow very dark and excoriating.

Pain in left ovarian region, extending to the groin, then down the thigh and leg. Entirely relieved by Ovi-gall-p.$^{cm}$.

Aching in the right ovarian region.

*Chest and Heart.*—Stitching pains constantly in the region of the heart, as if the left heart was too narrow, and smaller than the right, after CMM.

Dull pain from the heart to the left ovary, then extending all over the stomach and belly, after CMM.

Mrs. P. had suffered with her heart for many years, when under mental or physical excitement, and finally it took the form of congestion of the heart; the usual dull, heavy, aching feeling was supplemented by a numb, cold feeling internally, like a cold stone. She had a severe attack and at eight P. M. received a dose of Ovi-gall-p.$^{cm}$. She said: "In fifteen minutes after taking the medicine, I was taken with a sharp pain in the region of the heart, which passed directly downward as if into the bowels, and I had to fly to the closet, and passed a large volume of blood. Afterward I felt much better, slept splendidly, and woke without any of that annoying feeling at the heart. A few days after I got in a violent passion, but had no return of the old trouble. Awoke next morning with a feeling exactly like a bee sting in the heart or in that region, and have felt well since (Boardman)."

Mr. B. Heart feels heavy and dull and numb, as the foot does when asleep. Has had these symptoms for many years at various times; took Ovi-gall-p.$^{cm}$. In ten minutes was entirely relieved and symptoms have never returned.

Great uneasiness with sense of heat and heavy weight in region of the heart (received a few pellets of Ovi-gall-p.$^{cmm}$); soon after had a sweetish taste like blood in my mouth, at the same time the heart suffering began slowly to diminish. In about fifteen minutes my left nostril began to bleed bright red blood, about a teacup half-full. I had many years previously nose-bleed before menses. Next day my heart, body, mind, and whole life are light, and I feel perfectly well. (Boardman.)

Mrs. P. felt a severe thump or blow in the heart.

Mrs. ——. Heart feels heavy, dull, and numb as the foot feels when asleep. These symptoms she has had for many years. After Ovi-gall-p.$^{2c}$ she immediately began to get relief, and in ten minutes they all left and have never returned.

*Back and Neck.*—Backache, with feeling as though a vertebra had dropped out of the lumbar region, and if the spine was tied together with strings.

Pain in sacrum. (Severe.)

Pain in every vertebra of spine.

*Stiff Neck.*—During convalescence from typhoid fever he was continually complaining of his back in the region of kidneys; suffers very much. On stooping a sharp pain passes through the spine, crosswise, which is terrible; is partially relieved when sitting or lying quiet for awhile. Great pains between two vertebræ in renal region, felt laterally or crosswise on rising from a seat or stooping forward.

Aching in sacral region.

*Arms.*—Bone pains in hands, with *great dread of motion.*

*Legs.*—Heavy aching in lower extremities. (Ballard.)

*Skin.*—Skin discolored; dark.

*Measle*-like spots on her limbs, spreading to her chest, abdomen, and left forearm, without itching or any irritation, after some time a bran-like desquamation.

*Generalities.*—GROUP, in proving. Occipital headache; stiff neck; confusion of mind.

Pain in every vertebra.

Intolerance of bands on wrists, arms, waist, or garters.

Sensation of severe cold; sneezing; catarrh; cracked lips; great dread of motion ; watery leucorrhœa, not as acrid as before taking the powders.

Languor following or accompanying malaria, in hopeless, tearful people ; with inert mental condition, or those who appear worn out by mental strain or intellectual labor.

Great debility ; losing flesh.

Pain in the epigastric region, passing down the median line of the body ; then in left ovarian region, then in left hip, then down posterior side of left thigh to hollow of knee. At the same time a sense of great crookedness in left side, and especially in left leg, followed by a menstrual flow which continued about fifteen minutes.

Any exercise or walking brought on severe pain in left ovary and leg to feet, incapacitating her entirely. Cured by Ovi-gall-p.$^{cm}$.

Cannot bear the least pressure of her clothes ; has to have all suspended from her shoulders, and cannot wear corsets.

Debility, with great dread of motion, with mental depression and hopelessness.

Great prostration, and so weak she cannot attend to her household affairs ; exceedingly low-spirited ; no appetite.

Since menstruating, she says if she keeps quiet, walks, or stands a very little, lives well, amuses herself, she feels perfectly well. But a little extra effort or exercise in standing or walking breaks her down with the pains in the left ovarian region, thigh, and knee, followed by melancholy. Since taking Ovi-gall-p.$^{cmm}$, she has walked miles, worked steady and hard at house-cleaning, and had no returns of the pains or weariness.

Tired all the time—when getting up and when retiring.

Sensation as if she was too heavy for the bed, and that unless it is supported it will break down.

### Characteristics.

Griping cramps and diarrhœa coming on suddenly.

*Fever.*—Griping chills.

*Sleep.*—Lay awake all night as is usual with her before menses.

Sleepy all the time; can sleep any time night or day; is tired all the time.

Between ten A. M. and three P. M. was taken suddenly with intense drowsiness; sometimes overcoming the will to keep awake.

Especially sleepy during menstruation.

During sleep, and immediately on going to sleep, the whole body jerks.

At the suggestion of the late Dr. Baruch, who thought very highly of these preparations, I combined the three in one, naming it Ovi-gallinæ-pellicula-comp., or abbreviated O. G. P. comp., and I believe it will be found very valuable in affections of the heart and the uterus.

